# Supplementary material for: Biases in neural population codes with a few active neurons
Source: PLoS Comput Biol. 2025 Apr 11;21(4):e1012969. doi: 10.1371/journal.pcbi.1012969 (PMC12054897; doi:10.1371/journal.pcbi.1012969)
Supplement: Attachment — Submitted filename: reply_SK.pdf [file pcbi.1012969.s001.pdf]

Dear Editor,

We would like to thank you and the reviewers for their comments. In response we made the following amendments that concern all reviewers:

- Clarification of the text, including considerations for experimental verification of the findings.
- To demonstrate the wider applicability of our results, we added a panel to Figure 4 showing the bias in a long 1D array.
- To clarify the dependence on noise, we added a panel to Figure 3 showing the dependence explicitly.
- We added a reference to Yarrow & Series, and to Westheimer.

We hope that the paper now satisfies the reviewers.

Reviewer #1:

(...) There is no attempt to analyze things mathematically, which may in fact not be feasible.

We would like to emphasize that we do derive a general approximation for the bias in the Bayesian least square estimator (complementary to a method to calculate the biases in ML decoders presented in our earlier paper, Keemink &MvR 2018b). We have attempted to find a more precise approximation for the Bayesian estimator, but have not found a satisfactory approximation.

Reviewer #2:

(1) The theoretical analysis does not provide deeper insights into the simulation results. The approximate method proposed at the end of the paper, regardless of its validity, still leads to expressions containing integrals. These do not directly reflect the influence of key factors discussed earlier, such as tuning curve shape, tuning width, noise level, and neuron number, on the bias. (2) The paper lacks in-depth analysis of the simulation results. While the simulations are reported, there is insufficient exploration of the underlying mechanisms. For instance, the paper mentions that Von Mises tuning only results in attractive bias, whereas rectified cosine tuning exhibits three bias tendencies (repulsive, attractive, and bi-phasic). However, the authors only analyze the cause of the attractive bias, leaving the other two phenomena unexplained.

We did attempt to find intuition behind the findings, but have not been able to find it apart from the attractive case. Our approximation method reveals that indeed the dependence of the bias on tuning curve shape is non-trivial. Yes, the approximation contains an integral; we have not been able to find good approximations of it (see above). Additionally, as we note in the discussion, the 'approximation is efficient as it is just a single integral (per stimulus) and can be carried out with standard integration routines'. Finally, the analytical methods we consider here and in Keemink et al (2018) also provide evidence that our results are not merely numerical artifacts but a real property of the systems + decoders (see also our response to reviewer 3 about numerical artifacts and decoding distributions). We now include the above points in the discussion.

(3) Some of the paper's descriptions are imprecise. For example, on page 6, it states: "For intermediate thresholds ($c \approx 0.5$), the bias is negative, and at a given threshold, the curves in the figure are spaced out equally in the vertical direction, in other words, the bias is proportional to the noise." I disagree with the claim that the curves are equally spaced. The authors neither provide simulation evidence of the linear relationship between bias and noise nor offer a theoretical justification. This statement feels more like an unsubstantiated assumption.

This was unclear. The behaviour is only approximately linear in some regimes, but in most regions it is not. We now added a figure panel showing the scaling with noise explicitly (Figure 3B). We have also made improvements to the paper's text throughout.

(4) The figures are poorly constructed. Most of the figures fail to clearly convey their intended meaning. For instance, in Figure 6, all approximate theoretical results are represented by black dashed lines without distinctions in thickness or color, making it impossible for readers to compare theoretical and simulation results.

We have redone this figure and made additions to several other figures (see other comment responses), hopefully clarifying the data.

Conclusion:

While the paper's premise and some of its simulation findings are novel, it falls short of being a polished scientific paper. At best, it reads as a report summarizing a set of simulation results. The authors need to conduct a more thorough analysis of the observed phenomena and significantly improve the clarity of their figures and descriptions to enhance the paper's readability and scientific rigor.

Reviewer #3:

Overall, the paper makes a worthwhile contribution to the literature, although in many cases it appears the bias is so tiny for both ML and Bayesian estimators (eg Figs 1-4) that I found myself wondering if this level of bias is something that is worth caring about. It would be helpful if the authors could add some comments about what they consider to be a realistic setting (eg for tuning curve width and noise level), and whether they expect the bias they observe to have any meaningful behavioral consequences.

(see also response to point 2). We agree that that the bias is small. However, with enough trials it should always be possible to average away the variance component. As the new panel in Figure 4 shows (Figure 4B), the size bias is comparable to effects shown in hyper-acuity, so might be measurable. We have extended the Discussion on experimental considerations/predictions.

I found two significant shortcomings that I think should be addressed before the paper is suitable for publication.

1) Circular variables. The paper repeatedly invokes the Gaussian distribution, but it focuses primarily on a setting with circular variables, where the (standard) Gaussian distribution cannot be applied. It may be the case that the posterior distribution is narrow and can be well approximated locally by a Gaussian in many settings, but it is still not technically Gaussian. So the authors should take more care with the appropriate treatment of circular variables. Similarly, the mean is not well defined for circular variables. (...)

We thank the reviewer for this insight and checked our simulations. Because the posterior distributions are narrow and we consider encoded angles close to 0 (and we avoid 'wrap-around' effects), there is no distinguishable difference between traditional and circular statistics, see the figure below. It shows the bias of the Bayesian decoder for two different shape parameter settings. There is no difference between them. We have added a note on this to the manuscript.

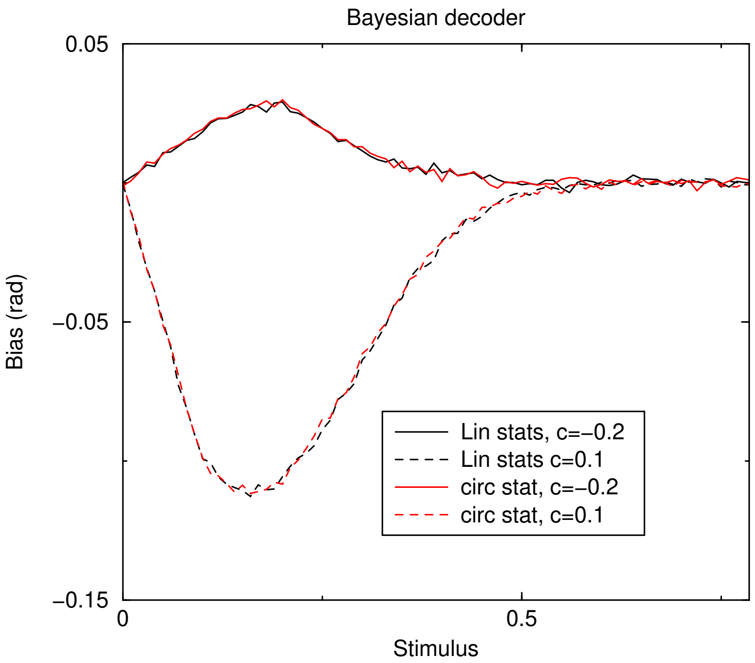

2) Variance. The paper focuses primarily on estimator bias; it considers variance only in Figure 5, using a single choice of tuning curve and noise level. However, I found myself thinking that in the case of the narrow tuning curves shown in Fig 2, the effects on variance might be far larger than those on bias.

(It would also be nice to see if the Bayesian estimator achieves lower variance than the ML estimator, since we have already seen that in some cases it exhibits higher bias). Overall, it seems the paper would be more substantial if the paper gave a more equal treatment of estimator variance (or even MSE), which I suspect might be more interesting than bias in some cases.

These are interesting questions.

1. Variance of the decoder has been studied extensively in the past (but bias has not). Hence we focus on the bias here. The variance of the ML is not uniformly higher than the Bayesian. The plot below is directly from the Dayan and Abbott book:

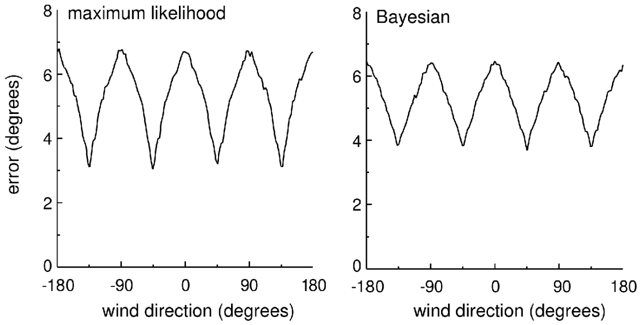

Figure 3.7: Maximum likelihood and Bayesian estimation errors for the cricket cercal system. ML and Bayesian estimates of the wind direction were compared with the actual stimulus value for a large number of simulated firing rates. Firing rates were generated as for figure 3.5B. The error shown is the root-mean-squared difference between the estimated and actual stimulus angles. (Adapted from Salinas and Abbott, 1994.)

2. The std.dev. of the decoder is in our study at least a factor 3-4 bigger than the bias (see for instance the histograms). This means that experiments would need to average over a substantial number of trials to measure the bias, but it would certainly seem possible. We have added these considerations to the discussion.

Abstract: "codes can also display biases" - this strikes me as non-standard usage of the word "bias". The technical definition of bias applies to estimators not "codes". (It's not clear to me what it would mean for a code to be biased; rather the estimators based on this code could be biased or unbiased.)

This was indeed sloppy. Corrected.

pg 2: "minimizing the likelihood" -> "maximizing the likelihood".

Corrected.

pg 2: "this is the mean of the distribution". -> "... posterior distribution".

Corrected.

pg 2: "The Bayesian decoder" This estimate (also known as "Bayes least squares") is perfectly fine in general terms, but it's worth noting that this formula is not applicable as written to circular variables like orientation. Unfortunately, the standard arithmetic mean isn't applicable to circular data. You could take the circular mean, which can be defined using complex numbers, although in this case the distance being minimized is slightly different than the Euclidean mean.

See above.

Fig 1A: This plot is relatively difficult to grok, and obscures the amplitude of the tuning curves. I would prefer to see the tuning curves plotted with "expected response" on the y axis and orientation theta on the x axis. The current plot lacks axis labels, so it's unclear what the 5 circles correspond to. (It would be helpful to label the tuning curves for different neurons, eg "neuron 1", "neuron 2", etc).

We replaced this panel with a more traditional plot. We also now state how many neurons will be simultaneously active.

Fig 1B. The caption says "Left: The estimate distribution for a few trials". But these look like posterior distributions (or perhaps likelihoods), not estimates. (I would think "estimate distribution" refers to a distribution of estimates, which is shown on the right). The estimate for a few trials would just be a few points. If these are likelihoods or posteriors, the x axis label should be "stimulus" not "stimulus estimate". (In this case the peak of each curve would be the ML or MAP estimate).

This was indeed unclear. Clarified.

Right side of Fig 1B, why is there a big spike in the histogram for the ML estimate? It's not obvious to me that this should occur -- is it possible there's an error due to finite gridding of the likelihood?

We have convinced ourselves this is not a numerical artefact and have seen in it multiple implementations. including our exact method (Keemink &MvR 2018b). We now mention this in the text. The graph below show that this occurs in both simulation and analytics. The intuition is perhaps that the maximum of a distribution is more robust than the mean of a distribution.

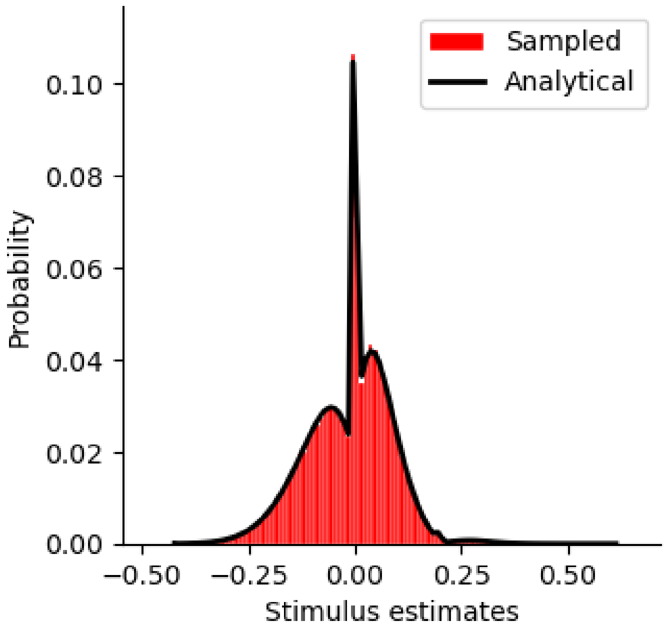

Fig 1B caption: "A Bayesian estimate extracts the mean of the distribution".

Once again, this is not quite correct for circular variables. (The cost function minimized by the circular mean is not the standard arithmetic mean, as noted above).

See above.

Fig 1B caption: "the arrow indicates the average estimate, i.e. the bias." Sorry, something is wrong here. The black arrow looks nowhere close to the mean of the black histogram. How can this be the average estimate? There's also a problem that the red arrow is not visible against the red histogram.

Thank you for spotting this. We have revised the figure and also removed the arrows.

Fig 1C: Y axis needs units. (Presumably the bias here is shown in units of radians?) Same comment applies to other figs showing bias (eg Fig 2, Fig 3, etc).

Now added (We note that formally radians are dimensionless SI units).

Fig 1C caption: "away from the preferred stimuli". Would be clearer to say "away from the preferred stimuli of the four neurons in the population."

Excellent suggestion. Modified.

Pg 4, "Emergence of Bias" section. Can the authors give any insight into why the estimate is biased here? Is it because only one neuron is active at the 4 cardinal orientations? (It's a little bit hard to tell from the tuning curves in Fig 1A if this is the case or not).

In this section there are always 2 or 3 neurons active. Only when the threshold is high ($c > 0$) there are stimuli for which only one neuron is active. This is worked out in the subsequent section and clarified.

Fig 2 and the next section do examine how tuning curve width affects the bias, but the bias ends up feeling mysterious here. If the tuning curves overlapped more I would expect this bias to go to zero. (What happens if the noise goes to zero -- does the bias disappear or is it still present? It seems the authors address this in Fig 3, but I didn't see them explicitly address whether bias goes to zero in the limit sigma -> 0.)

The following section gives an explanation for why there should be an attractive bias in the case of narrow tuning curves, but it still seems mysterious that there is a bias when the tuning curves are broad. Can the authors provide any intuition for why there's a bias in this case?

The bias for narrow tuning remains in the zero noise limit (at least for the rectified cosine tuning). The bias for wider tuning is noise dependent. We now show this noise dependence in a new panel of Figure 4.

pg. 4: "The likelihood becomes Gaussian". Again, this is not quite right for circular variables.

See above.

pg. 4: "However, when there are just a few noisy neurons active this is no longer true. For instance, Figure 3.7 in the textbook by Dayan and Abbott (2001) shows a subtle difference in the variance of the ML and Bayesian decoders, hinting at a non-Gaussian posterior distribution. Also here the distribution of estimate is non-Gaussian, Fig.1B right." Rather than focusing only on difference in the variance of the estimators, it might be helpful to remind readers why the ML estimate is not the same as the Bayesian estimate (even when the prior is uniform): it's because the mode of the posterior (which is also the mode of the likelihood) is not necessarily equal to the mean of the posterior. And the mean (but not the mode) of the posterior achieves minimum MSE. So in cases where the posterior is not symmetric, the mean and mode can be in different locations.

Thank you, that is much clearer. Text modified.

pg 6: "First we increased the number of neurons without changing any of the tuning curve properties,

I'm not sure what this means. Do you mean you replicated the existing tuning curves so there are now multiple copies of each of the 4 canonical tuning curves? Or you added tuning curves with the same shape but with different preferred orientations to create a population that tiles?

The latter, the preferred orientations were equally spaced. Text clarified.

pg 6. "bias only depends weakly on the signal-to-noise ratio of the neurons, consistent with the above observations." This seems to be contradicted by Fig 3, which shows (for both kinds of tuning curves) that bias grows much smaller with reduced noise. Can you clarify?

This was a confusing statement. We decided to remove it.

pg 7: typo: "variance of the decoder varies across with the stimulus"

Corrected.

Have the authors made all data and (if applicable) computational code underlying the findings in their manuscript fully available?

We now made code available at `https://github.com/vrossum/bias24`
