# Supplementary material for: Biases in neural population codes with a few active neurons
Source: PLoS Comput Biol. 2025 Apr 11;21(4):e1012969. doi: 10.1371/journal.pcbi.1012969 (PMC12054897; doi:10.1371/journal.pcbi.1012969)
Supplement: Attachment — Submitted filename: reply_r2.pdf [file pcbi.1012969.s002.pdf]

March 5, 2025

Dear Editor,

We would like to thank you and the reviewers for their comments. Reviewer #3 noted:

I have only one lingering comment, which is that I would encourage the authors to provide a bit more extensive discussion of the implications of using Gaussian distributions to describe circular variables. (...)

We have now clarified this. Indeed the term linear statistics was confusing. We now use 'standard statistics'. We also explain in more detail how we checked that standard statistics sufficed.

We hope that the paper now satisfies the reviewers.

For all authors

Mark van Rossum
